# Psilocybin-induced modulation of visual salience processing

**DOI:** 10.1093/nc/niaf060

**Published:** 2025-12-26

**Authors:** Stephanie Muller, Federico Cavanna, Laura Alethia de la Fuente, Nicolás Bruno, Tomás Ariel D'Amelio, Carla Pallavicini, Enzo Tagliazucchi

**Affiliations:** National Scientific and Technical Research Council (CONICET), Godoy Cruz 2290, C1425 Cdad. Autónoma de Buenos Aires, Argentina; Institute of Applied and Interdisciplinary Physics and Department of Physics, University of Buenos Aires, C1428 Cdad. Autónoma de Buenos Aires, Argentina; National Scientific and Technical Research Council (CONICET), Godoy Cruz 2290, C1425 Cdad. Autónoma de Buenos Aires, Argentina; Institute of Applied and Interdisciplinary Physics and Department of Physics, University of Buenos Aires, C1428 Cdad. Autónoma de Buenos Aires, Argentina; National Scientific and Technical Research Council (CONICET), Godoy Cruz 2290, C1425 Cdad. Autónoma de Buenos Aires, Argentina; Institute of Applied and Interdisciplinary Physics and Department of Physics, University of Buenos Aires, C1428 Cdad. Autónoma de Buenos Aires, Argentina; Institute of Cognitive and Translational Neuroscience, INECO Foundation, Favaloro University, Marcelo Torcuato de Alvear 1632, C1021 Cdad. Autónoma de Buenos Aires, Argentina; National Scientific and Technical Research Council (CONICET), Godoy Cruz 2290, C1425 Cdad. Autónoma de Buenos Aires, Argentina; Institute of Applied and Interdisciplinary Physics and Department of Physics, University of Buenos Aires, C1428 Cdad. Autónoma de Buenos Aires, Argentina; Frontlab, Paris Brain Institute (ICM), Hôpital Pitié, 47 Bd de l'Hôpital, 75013 Paris, France; National Scientific and Technical Research Council (CONICET), Godoy Cruz 2290, C1425 Cdad. Autónoma de Buenos Aires, Argentina; Institute of Applied and Interdisciplinary Physics and Department of Physics, University of Buenos Aires, C1428 Cdad. Autónoma de Buenos Aires, Argentina; Centre de Recerca Matemàtica (CRM), Carrer de l'Albareda, 08193 Bellaterra, Barcelona, Spain; National Scientific and Technical Research Council (CONICET), Godoy Cruz 2290, C1425 Cdad. Autónoma de Buenos Aires, Argentina; Institute of Applied and Interdisciplinary Physics and Department of Physics, University of Buenos Aires, C1428 Cdad. Autónoma de Buenos Aires, Argentina; Integrative Neuroscience and Cognition Center, University of Paris Cité, 45 Rue des Saints-Pères, 75006 Paris, France; National Scientific and Technical Research Council (CONICET), Godoy Cruz 2290, C1425 Cdad. Autónoma de Buenos Aires, Argentina; Institute of Applied and Interdisciplinary Physics and Department of Physics, University of Buenos Aires, C1428 Cdad. Autónoma de Buenos Aires, Argentina; Latin American Brain Health (BrainLat), Universidad Adolfo Ibáñez, Av. Diag. Las Torres 2640, 7941169 Santiago, Peñalolén, Región Metropolitana, Chile; Center for Cognitive Neuroscience, Universidad de San Andrés, Vito Dumas 284, B1644BID Victoria, Provincia de Buenos Aires, Argentina

**Keywords:** psilocybin, salience, attention, eye tracking, perception

## Abstract

Psychedelic compounds significantly reshape conscious perception, yet the implications of these alterations for complex visual-guided behaviors remain poorly understood. We investigated how psilocybin modulates visual salience processing during natural scene perception. Twenty-three participants completed eye-tracking tasks under self-blinded low and high doses of psilocybin, in a naturalistic design with experimental conditions unknown to participants and researchers. Subjects viewed natural scenes while their gaze patterns were recorded and analyzed in relation to normative computational saliency maps generated using a deep learning model of visual attention. Results revealed increased fixation on salient image regions and reduced inter-fixation distance under the high-dose condition, suggesting heightened sensitivity to visual salience and more localized gaze behavior. The Shannon entropy of fixations on high-saliency regions indicated a more exploratory and less predictable visual scanning of the images. Complementary resting-state electroencephalography recordings showed broadband spectral power reductions and increased Lempel-Ziv complexity, with delta power negatively correlating with salience metrics. These findings indicate that psilocybin induces a shift in attentional dynamics, altering gaze behavior, and salience processing during natural scene perception.

## Introduction

Serotonergic psychedelics are notable for their ability to induce profound alterations in sensory perception (Nichols 2016, van Elk and Yaden 2022). In the visual domain, psychedelic tryptamines such as psilocybin and dimethyltryptamine induce low-level alterations, including changes in the color and texture of objects and the appearance of geometric distortions (Preller and Vollenweider 2016, Kometer and Vollenweider 2018, Aday et al. 2021). Despite these widespread effects, there are relatively few studies investigating how visual alterations impact complex behaviors that depend on the integration of multiple cognitive functions (Bălăeţ 2022). One particularly underexplored area is the impact of psychedelics on attentional processes involved in integrating visual information for object recognition in natural scenes.

Humans navigate their environment by continuously sampling visual information through a sophisticated pattern of eye movements, including saccades, fixations, and microsaccades, collectively enabling the dynamic exploration and processing of visual scenes (Findlay and Gilchrist 2003). The neural and temporal costs associated with the serial nature of fixations require mechanisms for selecting certain aspects of the image over others (Gottlieb et al. 2014). Bottom-up prioritization is driven by visual salience and depends on low-level image features such as specific colors, shapes, contrast, and motion. Fixation patterns are also modulated by top-down factors including task-demands, higher-order cognitive processes, and individual variations. Bottom-up and top-down influences interact dynamically to guide visual attention, balancing sensory-driven responses with goal-directed control of gaze (Fecteau and Munoz 2006, Tatler et al. 2011).

Psychedelic substances can influence this process at multiple instances in parallel, with an overall uncertain effect. Perceptual distortions may affect the identification of low-level information in the visual scene, interfering with the formation of saliency maps and basic feature detection. These alterations include changes in contrast sensitivity, color perception, and edge detection, which could fundamentally alter how visual information is processed in early visual areas (Kometer and Vollenweider 2018). Psychedelics have also been shown to broaden visual perceptual bandwidth, as evidenced by increased prepulse inhibition (Quednow et al. 2012, Vollenweider et al. 2007). Impaired evaluation of sensory signal accuracies can result in a more disorganized or entropic exploration of the visual scene (Carhart-Harris et al. 2014). Additionally, changes in attention allocation and cognitive control mechanisms might contribute to more erratic scanning patterns and modified fixation strategies (Carter et al. 2005, Quednow et al. 2012, Bălăeţ 2022).

The present study investigated the effects of psilocybin on visual salience processing during natural scene perception. Following a randomized self-blinded protocol conducted in ecologically valid settings, participants completed two dosing sessions—one low-dose and one high-dose of psilocybin mushrooms on separate days and were subsequently presented with natural scenes for brief time intervals. The sequence of gaze fixations was recorded using an eye-tracking device, informing how the subjects prioritized specific regions of the images. In combination with self-reported psychometric data, resting-state electroencephalography (EEG) was recorded before and after dosing to obtain spectral markers of subjective effect intensity, and to provide complementary neural correlates to the eye-tracking measures of psilocybin-induced modulation of visual salience processing.

We examined four primary hypotheses regarding the impact of psilocybin on visual perception and attention. First, we hypothesized changes in salience associated with gaze fixations under psilocybin, in relation to a normative model of spatial salience calibrated with data from individuals in a normal state of consciousness. Second, we hypothesized increased entropy in visual exploration patterns across high salience regions, quantified as the Shannon entropy of the distribution of fixations across the clusters of highest salience in each image, where clusters were defined as contiguous regions above a saliency threshold. Third, we anticipated a broadband reduction in EEG spectral power, hypothesized to correlate with the behavioral measures outlined in our initial predictions. Lastly, based on our previous findings regarding esthetic perception (Muller et al. 2023), we predicted specific alterations in general visual exploration strategies, particularly a more localized exploration of the visual scene.

## Martials and methods

### Participants

Twenty-three participants [four females, 31 ± 4 years, 72 ± 15 kg (mean ± SD)] were recruited through word of mouth and social media advertising. To participate, individuals were instructed to contact the provided number *via* WhatsApp and subsequently received a phone call from the researchers. During the call, the researchers provided a brief explanation of the details and purpose of the experiment, as well as the inclusion and exclusion criteria. Participants were then given a full written explanation of the study and a copy of the informed consent form. To determine eligibility, all participants underwent a psychiatric interview to screen for exclusion criteria, which are detailed in the supplementary material and summarized below. After this screening, participants and researchers agreed on a date for the start of the experiment. Participants reported having 15 ± 13 prior experiences with serotonergic psychedelics, of which 3.1 ± 2.4 were considered challenging [mean ± SD]. All participants had normal or corrected-to-normal vision.

This study was conducted in accordance with the Declaration of Helsinki and approved by the Research Ethics Committee at the Universidad Abierta Interamericana (Buenos Aires, Argentina), protocol number 0-1068. All participants gave written informed consent and received no financial compensation for their participation in the experiment. All data was collected from the participants in natural settings, i.e. those chosen by the participants without intervention from the researchers. The researchers did not provide nor administered psilocybin to the participants, nor instructed them in any way concerning drug use.

### Inclusion and exclusion criteria

Participants were required to have at least two prior experiences with a dose equal to or exceeding 3 g of dried psilocybin mushrooms. To participate in this research protocol, subjects volunteered to partake in a series of tests under the effects of psilocybin mushrooms and in the presence of four members of the research team. Subjects who consumed serotonergic psychedelics during the 15 days prior to the dosing day were not included in the study. The same applied for all psychoactive substances (including alcohol, caffeine, and tobacco) for a period of 24 h prior to the dosing day. To participate in the experiment, subjects declared their willingness to abstain from using psychedelics between measurement sessions. Drug use prior to the study was assessed by self-report; no biochemical tests were performed.

Subjects who fulfilled the Diagnostic and Statistical Manual of Mental Disorders (DSM-5) criteria for the following disorders were excluded from the experiment: schizophrenia or other psychotic disorders, type 1 or 2 bipolar disorder (including first- and second-degree relatives), personality disorders, dissociative identity disorder, post-traumatic stress disorder, substance abuse or dependence in the past 5 years, depressive disorders, recurrent depressive episodes, obsessive-compulsive disorder, generalized anxiety disorder, dysthymia, panic disorder, bulimia or anorexia, as well as subjects with a history of neurological disorders. Pregnant women and subjects under psychiatric medication of any kind were excluded. Subjects exhibiting potential dysfunctional states as measured by the Depression Anxiety Stress Scale (with scores > 4 for depression, > 3 for anxiety, and >7 for stress) were subsequently reviewed by the clinical interviewer for confirmation.

### Experimental design and setting

Experimental conditions (high-dose vs. low-dose) were a priori unknown to participants and researchers as subjects implemented a self-blinding procedure, following the design introduced by Szigeti et al. (2021). The experiment was divided into two parts, one corresponding to the a high-dose condition (3 g of ground and homogenized -mixed to ensure uniform psilocybin distribution- dried psilocybin mushrooms in gel capsules, provided by the participants) and one corresponding to a low-dose control condition (0.5 g of ground and homogenized dried psilocybin mushrooms mixed with edible mushrooms to match the weight of the gel capsules between conditions). The low-dose served as an active control. Chemical analyses of *Psilocybe cubensis* indicate ~ 1% total psilocybin + psilocin content by dry weight (range ~0.9%–1.3%; Goff et al. 2024). Thus, the 3 g high-dose corresponds to ~ 30–35 mg of active alkaloids, consistent with a moderate dose, while the 0.5 g low-dose corresponds to ~ 5 mg, within a threshold-to-low range. Conditions were separated by an interval of 1 month to attenuate potential tolerance effects. During interviews conducted after completion of the experiment, we confirmed that participants implemented the self-blinding assisted by a third party of their choice. In all cases, experiments took place in the comfortable setting of a house and in the presence of the participant and the team of researchers. During the high-dose session, two participants opted to discontinue the eye-tracking task. Consequently, they were excluded from subsequent analyses of eye-tracking data, although no adverse effects were reported.

### Self-reported scales and questionnaires

Two days before the dosing day for each condition, participants completed a set of self-reported questionnaires designed to assess various psychological traits. These included the State-Trait Anxiety Inventory (Spielberger 1983), the Big Five Inventory (John et al. 1991), the Tellegen Absorption Scale (Tellegen and Atkinson 1974), and the Short Suggestibility Scale (Kotov et al. 2004). Additional self-report scales were administered both before dosing and immediately after the acute effects, including the State-Trait Anxiety Inventory (Spielberger 1983), the Positive and Negative Affect Schedule (Sandin 1999), and the Psychological Well-being Scale (Luna et al. 2020). Before dosing, participants also completed assessments on expectations (EXP) and contextual factors related to prior psychedelic experiences (Haijen et al. 2018). Following the acute effects, participants completed the Mystical Experience Scale (MEQ) (MacLean et al. 2011) and the Altered States of Consciousness Scale (5D-ASC) (Studerus et al. 2010). In addition, participants rated subjective drug effects using a visual analog scale (VAS) at four time points during the dosing day, beginning 1 h after ingestion and continuing at hourly intervals until the effects subsided. A comprehensive description of all questionnaires used in the study is provided in the supplementary material.

### Stimuli

A total of 100 images were randomly subsampled from the Object and Semantic Images and Eye-tracking (OSIE) dataset (Xu et al. 2014), which originally comprised 700 images. The original OSIE dataset was deliberately designed with the objective of mitigating the biases that are commonly observed in existing image collections. In contrast to traditional datasets, which often feature a single dominant object in a central position, the images in OSIE are characterized by the presence of multiple dominant objects distributed across the scene. The images are diverse, spanning a wide range of object categories with strong semantic relevance. This design supports detailed analyses of attentional dynamics by incorporating both pixel-level details and object- and semantic-level attributes. The selected images had a resolution of 800 × 600 pixels and were displayed against a uniform grey background to ensure consistent viewing conditions. The same set of images was presented in both the high-dose and the low-dose conditions, ensuring that any potential influence of low-level visual features (e.g. luminance, color) was balanced across conditions. Nevertheless, individual images were not spatially homogeneous in terms of their low-level visual features (i.e. some regions could have higher luminance, color, etc.), which could have increased the saliency of specific regions independently of their semantic content.

### Eye tracking

The measurements described below were conducted as part of a broader study examining the effects of psychedelics on perception, creativity, language, and music production. Findings related to these domains will be presented in future reports. Prior to the task described in this study, participants completed another eye-tracking task involving free exploration of artworks from various periods and styles, with the results of this experiment detailed in a previous publication (Muller et al. 2023).

For the eye-tracking tasks, participants were seated 60 cm from a centrally positioned monitor, with their heads stabilized using a custom-made chin rest to minimize movement. The eye tracker was positioned between the participant and the screen. The display measured 31 × 17.5 cm and had a resolution of 1920 × 1080 pixels. During the presentation of visual stimuli, gaze coordinates were recorded in both spatial and temporal dimensions using the Gazepoint GP3 HD portable eye tracker, which operates at a temporal resolution of 150 Hz and achieves a visual angle accuracy of 0.5°–1.0°. Stimulus presentation and eye-tracking control were managed by a custom Python script developed using the open-source PsychoPy library (https://www.psychopy.org/).

**Figure 1:**
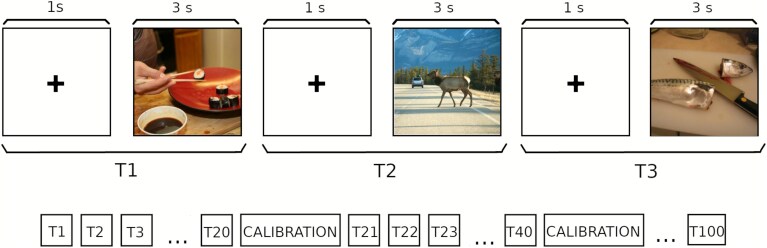
Overview of the eye-tracking task. Trials consisted of an initial calibration phase, followed by a first block (T1), consisting of a fixation cross displayed for 1 s and a 3 s stimulus presentation period, with this procedure repeated for 100 images with intermediate calibrations every 20 blocks.

The task reported in this study began 90 min after participants consumed capsules containing either a low or high dose of psilocybin. An overview of the experimental procedure is provided in Fig. 1. Each trial (T) commenced with a fixation cross displayed for 1 s, followed by a stimulus presentation lasting 3 s, during which participants were free to observe the image. The same stimuli were used across all experimental conditions, with their order randomized for each participant and condition. A standard five-point calibration was conducted after every 20th block to ensure that the eye tracking detected fixations accurately throughout the duration of the experiment.

### Statistical fixation metrics

Due to insufficient data quality, i.e. more than 30% of fixations either missing or recorded outside the screen for over 30% of the stimuli (suggestive of lack of engagement with the task), data from six participants was excluded from the present analysis. Furthermore, for each participant and condition, only images with less than 30% of fixations outside the boundaries were analyzed. This threshold corresponds to more than 2 SDs above the mean proportion of outside-screen fixations and applying it resulted in the exclusion of only a small and comparable proportion of trials across conditions. All fixations beyond the image boundaries were excluded from further analyses. No significant differences were found in the number of excluded fixations across experimental conditions.

A velocity-based algorithm (Engbert and Kliegl 2003) was employed to automatically identify fixations with minimum durations of 150 ms following the recording of gaze position coordinates (Manor and Gordon 2003). The mean horizontal (${x}_i$) and vertical (${y}_i$) positions of each fixation, along with the time of the *i*th fixation (*t_i_*) were then determined, with positions expressed in pixels and time in seconds. For each image, participant, and condition, the following metrics were calculated: the total number of fixations within the image (*N*), the mean distance between fixations (*ds*), and the mean time between fixations (*dt*). The following formulas were applied to compute these measures:


$$ ds=\frac{1}{N-1}{\sum}_{i=1}^N\sqrt{{\left({x}_{i+1}-{x}_i\right)}^{{}^2}+{\left({y}_{i+1}-{y}_i\right)}^{{}^2}} $$

$$ dt=\frac{1}{N-1}\sum_{i=1}^N\left({t}_{i+1}-{t}_i\right) $$

These measures were subsequently averaged across all images, yielding a mean value for each participant under each dosing condition.

### Visual saliency

To analyze the visual saliency, we first converted each image into a saliency map using DeepGaze II, a computational model of visual attention based on deep neural networks trained to predict human fixations on natural images. The model was pretrained on the SALICON dataset, which contains 10 000 diverse images annotated with mouse-tracking data, and subsequently fine-tuned on the MIT1003 dataset, composed of 1003 natural scene photographs viewed by human participants under eye-tracking for 3 s. Validation was conducted via 10-fold cross-validation on the MIT1003 dataset to ensure the model generalizes well to unseen images within the same domain (Linardos et al. 2021). The generated saliency maps reflect the likelihood of a region capturing visual attention. Each recorded fixation was then mapped to its corresponding saliency value from the respective image’s saliency map. The average saliency for each trial was calculated, and a final saliency value for each participant and experimental condition was obtained by averaging across all images. To examine how participants’ focus on salient regions evolved over time, we divided the 3-s viewing period into five equal intervals of 600 ms each and calculated the average saliency of the fixations within each subinterval.

### Shannon entropy in high salience regions

To quantify the predictability of fixation patterns in high saliency regions, we computed the Shannon entropy of fixation distributions within high-saliency areas. The saliency map was first binarized using a threshold, which was systematically varied in subsequent analysis to assess the robustness of the results across different saliency levels. The definition of this threshold and the range of values explored are detailed in the following paragraph. Connected clusters of the binary map were identified and treated as individual regions with high visual salience (see Fig. 4a). Each fixation was assigned to a high salience cluster based on its spatial location. The Shannon entropy was then calculated for the resulting distribution of fixations across clusters, using the formula:


$$ \mathrm{SE}=-\sum_{i=1}^k{p}_i\log \left({p}_i\right) $$

where ${p}_i$ represents the proportion of fixations within the *i*th cluster relative to the total number of fixations within the clusters and $k$ is the total number of clusters. Thus, higher entropy values indicate a more uniform distribution of fixations across the high salience clusters of the image.

This analysis only included images with more than one cluster to ensure the entropy calculation accurately reflected variability in fixation patterns. The analysis was performed across a range of thresholds to ensure the results were not biased by the chosen value. The lower bound of the explored range was defined as the threshold at which at least 70% of the images contained more than one cluster, while the upper bound corresponded to the point where the average number of clusters started to decline, indicating a loss of information about salient regions. For each threshold, entropy was compared between dosing conditions.

### Electroencephalography acquisition, preprocessing, and analysis

Resting state EEG data was recorded using a 24-channel mobile system (mBrainTrain LLC, Belgrade, Serbia; http://www.mbraintrain.com/) paired with an elastic electrode cap (EASYCAP GmbH, Inning, Germany; www.easycap.de). Twenty-four Ag/AgCl electrodes were positioned according to the standard 10–20 system at the following locations: Fp1, Fp2, Fz, F7, F8, FC1, FC2, Cz, C3, C4, T7, T8, CPz, CP1, CP2, CP5, CP6, TP9, TP10, Pz, P3, P4, O1, and O2. Reference and ground electrodes were placed at FCz and AFz, respectively. The wireless EEG DC amplifier (weight: 60 g; dimensions: 82 × 51 × 12 mm; resolution: 24-bit; sampling rate: 500 Hz; passband: 0–250 Hz) was securely attached to the back of the electrode cap, between electrodes O1 and O2. EEG signals were digitized and transmitted via Bluetooth to a notebook operated by the experimenter, who was positioned behind the participant. Participants were instructed to undertake 5-min recordings with their eyes closed, both 15 min prior to and 2 h after the administration of the dose. During these recordings, the subjects were asked to keep their eyes fully closed, relax all muscles, and avoid any physical tension.

EEG data was preprocessed using the Python MNE library (Gramfort 2013). A band-pass filter with a frequency range of 0.5–90 Hz was applied to the data to remove low-frequency drift and high-frequency noise. Additionally, a notch filter was employed to eliminate power line noise at 50 Hz and its harmonics. The filtered data were then segmented into fixed-length epochs of 2 s for subsequent analysis. Automatic epoch rejection was conducted using MNE’s autoreject algorithm (Jas et al. 2017), which identified and excluded epochs characterized by excessive noise. A manual inspection was conducted to ensure the quality of the data. Independent component analysis (ICA) was then applied to the cleaned epochs to identify components that were associated with artifacts such as eye blinks and muscle activity. This was subsequently corroborated by visual inspection. The remaining bad channels were interpolated, and the data was re-referenced to the average of all channels. Participants were excluded from EEG analyses when one of the conditions resulted in fewer than 50 valid epochs (<100 s). Three were removed, leaving 20 participants (≥75 epochs per condition). ICA removed on average 3.45 components in the low-dose condition and 2.80 in the high-dose condition. This resulted in 20 subjects for subsequent analysis, with a mean of 119 ± 22 epochs.

The power spectral density (PSD) was computed for each participant and experimental condition using the *MNE* implementation, applying the multitaper method. The minimum and maximum frequencies were set to 1 and 40 Hz, respectively. PSDs were averaged across epochs and channels to obtain a single value per 0.5 Hz and then logarithmically scaled. To assess dosing condition differences while correcting for multiple comparisons, we performed a cluster-based permutation test across frequencies using MNE’s *permutation_cluster_1samp_test* (5000 permutations, *P* < .05 at the cluster level)*.* Additionally, PSD data was aggregated into specific frequency bands: delta (1–4 Hz), theta (4–8 Hz), alpha (8–13 Hz), beta (13–30 Hz), and gamma (30–40 Hz) and averaged across epochs. This information was used to plot topomaps for each frequency band in each condition, and topomaps for the statistical test between conditions, identifying channels showing significant differences.

The complexity of broadband signals was assessed using the Lempel-Ziv lossless compression algorithm (Schartner et al. 2017, Timmermann et al. 2019, Cavanna et al. 2022, Pallavicini et al. 2021). This algorithm divides a binary string into non-overlapping, unique substrings. The greater the diversity within the string, the larger the number of substrings. The total count of these substrings is referred to as the Lempel–Ziv complexity (LZc). The initial step involved computing the instantaneous signal envelope for each epoch and channel, using the Hilbert transform. Following this, Z-score normalization and binarization through a median split were performed. The median split ensured an equal proportion of 1 and 0 s across all channels, thereby mitigating biases arising from unbalanced sequences. Finally, LZc was calculated for the binarized signal of each epoch and channel, and the values were averaged across epochs to obtain a single value per channel.

### Statistical analyses

Questionnaire results were compared between the dosing conditions using Student’s paired t-test, as implemented in Python’s Scipy library (https://scipy.org). We reported uncorrected *P*-values, and highlighted instances where *P*-values remained significant after applying the Benjamini–Hochberg false discovery rate (FDR) correction at α = 0.05. Corrections were not applied to instances where statistical independence could not be assumed, such as entropy results derived from multiple thresholds. Effect sizes were calculated using Cohen’s d for parametric tests and rank-biserial correlation (RBC) for non-parametric tests, with a detailed summary provided in Supplementary Table S1. Estimating the appropriate sample size was challenging due to the limited prior research on eye-tracking metrics in psychedelic studies. We estimated a sample size of 23 participants based on an expected medium to large effect size (*d* = 0.6), which is commonly observed in studies of psychedelics like psilocybin, with statistical power set to 0.8 and a significance level of 0.05, in a two-tailed, within-subjects design. To complement frequentist analyses, Bayesian statistics were employed to assess the relative evidence for the null and alternative hypotheses. We computed the Bayes factor (BF10) favoring the alternative hypothesis, using Python’s pingouin library (https://pingouin-stats.org) (Held and Ott 2018), with BF10 < 1/3 interpreted as supporting the null hypothesis. Non-parametric Wilcoxon signed-rank tests were used to compare eye-tracking measures and VAS scores across dosing conditions.

## Results

The experimental condition was correctly unblinded by participants in 42 out of 46 measurement days (91%), with this rate being identical for both the high-dose and low-dose conditions. Details on all self-reported scales and questionnaires can be found in the supplementary material (Supplementary Table S1), where effect sizes (Cohen’s d) and BF10 are also reported. As anticipated, the high-dose condition produced significant increases across all items of the MEQ30 and 5D-ASC questionnaires. The temporal evolution of the total VAS score is shown in supplementary Fig. S1.

### Statistical fixation metrics

**Figure 2:**
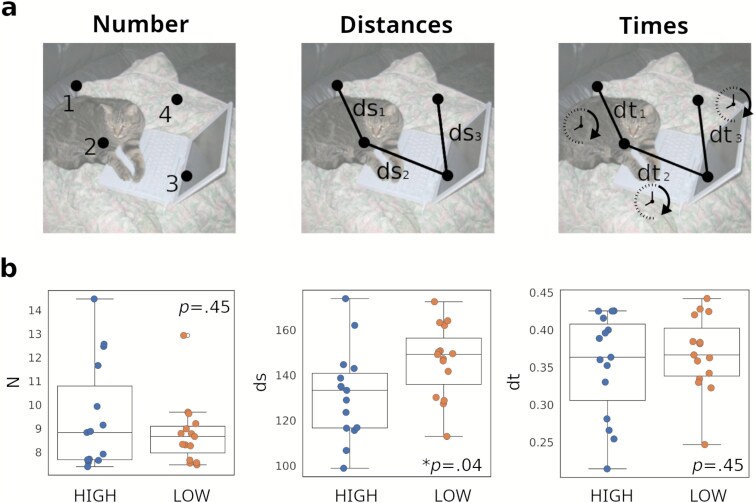
(a) Illustration of the statistical fixation metrics that were calculated for each trial, from left to right: number of fixations (*N*), distance between consecutive fixations (*ds*), and time between consecutive fixations (*dt*). (b) Values of the metrics *N*, *ds*, and *dt*, averaged across stimuli for each subject, and compared between the high-dose (HIGH) and the low-dose (LOW) conditions; the *P*-values (Wilcoxon signed-rank tests) are shown as insets. ^*^P < .05 (figures display boxplots with the interquartile range (IQR); the median is shown as a central line, whiskers extend to 1.5 × IQR, and individual participant data are overlaid as scatter points).

The total number of fixations (*N*), average distance between fixations (*ds*), and average time between fixations (*dt*), were computed for each stimulus and averaged over all stimuli for each subject and dosage condition, as detailed in the Methodology section. To assess differences between dose conditions, we performed non-parametric paired Wilcoxon signed-rank tests (Fig. 2b). The corresponding uncorrected (*N* = 3) *P*-values are reported in the figure. The analysis revealed a significant effect of dosing, with the metric *ds* exhibiting lower values under the high-dose condition. Effect sizes based on RBC are reported in Supplementary Table S2. As an additional control, we also examined the percentage of fixations outside the screen boundaries, which did not differ between high-dose and low-dose conditions. The corresponding plot is shown in Supplementary Fig. S2.

### Visual saliency

**Figure 3:**
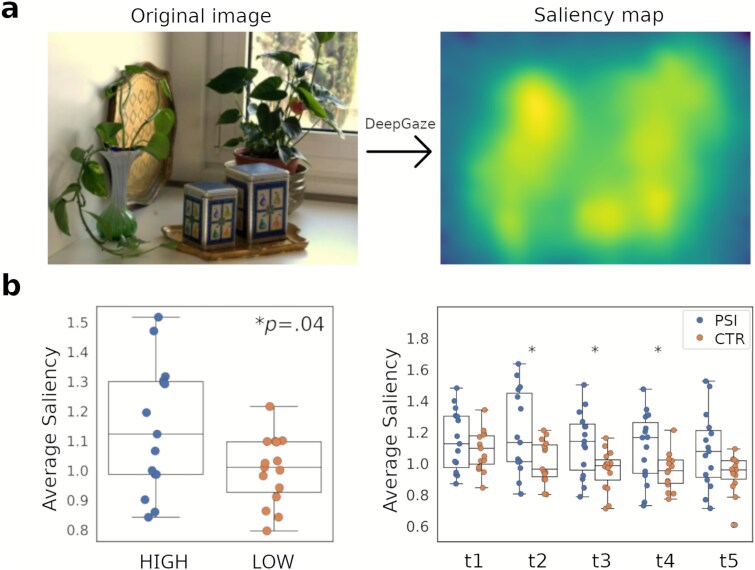
(a) Example of saliency map generation using DeepGaze II. (b) Average saliency values across stimuli for each subject, compared between the high-dose (HIGH) and the low-dose (LOW) conditions; left: overall average across fixations, with the *P*-value (Wilcoxon signed-rank test) shown as inset; right: averages calculated for five equal time intervals during the 3-s viewing period ( ^*^P < .05. Figures display boxplots with the interquartile range (IQR); the median is shown as a central line, whiskers extend to 1.5 × IQR, and individual participant data are overlaid as scatter points).

As described in the Methods section, normative saliency maps were generated using *DeepGaze II* to quantify the visual salience associated with the subjects’ fixations (Fig. 3a). The average saliency values across all images were compared between the high-dose (HIGH) and low-dose (LOW) conditions. The high-dose condition showed significantly greater saliency compared to the low-dose condition. To further examine temporal dynamics, the 3-s viewing period was divided into five equal 600 ms intervals. Three of these five intervals showed significantly greater saliency in the high-dose condition compared to the low-dose condition: t2 (*P* = .026), t3 (*P* = .035), and t4 (*P* = .026) (Fig. 3b). Both uncorrected and FDR-corrected *P*-values are provided in Supplementary Table S3. Effect sizes based on RBC are reported in Supplementary Table S2.

### Shannon entropy in high salience regions

**Figure 4:**
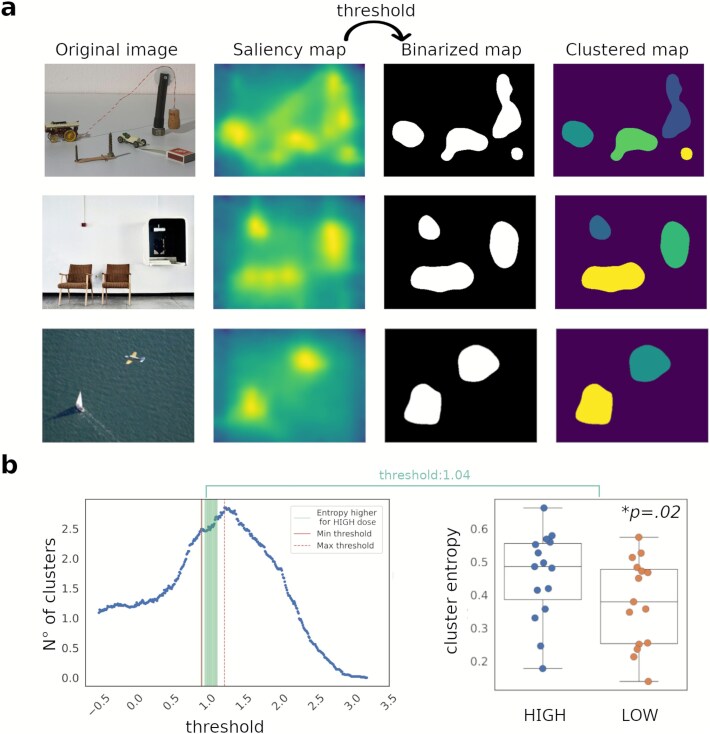
(a) Examples of high saliency region clusterization: original image, saliency map, binarized map, and clustered map are shown for three different stimuli. (b) Left: number of clusters as a function of the threshold (the vertical lines mark the selected range of thresholds used for entropy calculations, while the green lines indicate thresholds where entropy was significantly different); right: example of entropy values for a specific threshold within the selected range, showing significantly higher entropy for the high-dose (HIGH) condition (the figure displays a boxplot with the interquartile range (IQR); the median is shown as a central line, whiskers extend to 1.5 × IQR, and individual participant data are overlaid as scatter points).

The calculation of Shannon Entropy of fixation distributions across high-saliency visual clusters, as described in the Methods section, is illustrated in Fig. 4a. The number of clusters detected in the binarized saliency maps varied as a function of the threshold, as shown in the left panel of Fig. 4b. To ensure a robust calculation of entropy, we selected a range of thresholds (marked by red lines). Green vertical lines indicate all thresholds within this range where entropy values were significantly different between conditions, with higher entropy consistently observed for the high-dose psilocybin (HIGH) condition compared to the low-dose (LOW) condition. The right panel of Fig. 4b illustrates this result for a specific threshold within the selected range.

### Electroencephalography spectral power and Lempel Ziv complexity

**Figure 5:**
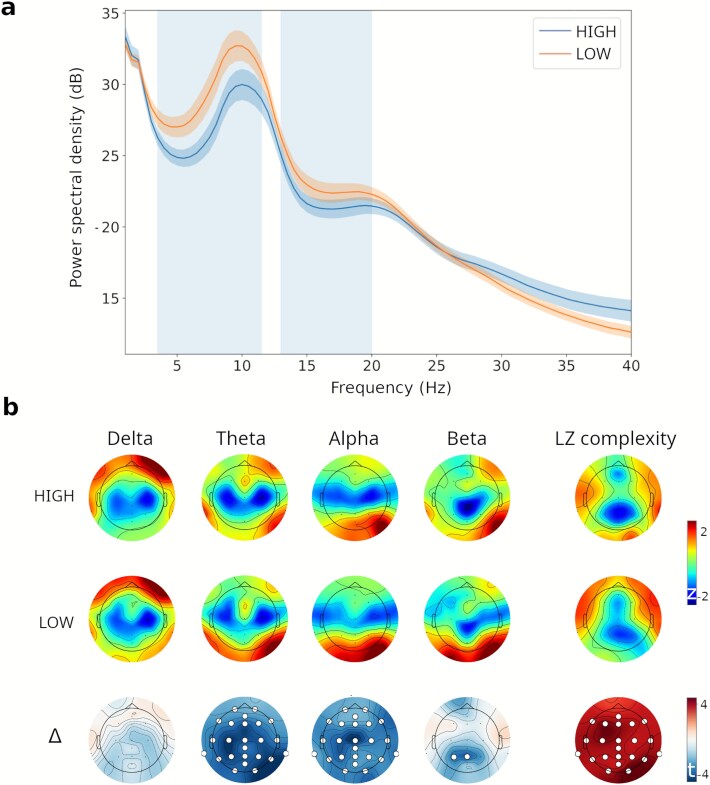
(a) Logarithmic PSD averaged across all channels, with shaded areas indicating frequency clusters where the cluster-based permutation test revealed significant differences between conditions (*P* < .05), with reduced power in the high-dose condition. (b) Topographic maps of spectral power across frequency bands and LZc for both dose conditions, along with topographic maps of t-values from statistical tests comparing conditions.

We compared the logarithmic PSD between dose conditions, averaged across all channels, using a cluster-based permutation test (Fig. 5a). Two significant clusters were identified: one spanning 3.5–11.5 Hz (*P* = .017) and another spanning 13.0–20.0 Hz (*P* = .037), both showing reduced spectral power in the high-dose condition relative to the low-dose condition. These clusters correspond to the upper delta and theta to low alpha range, and the high alpha to beta range, respectively.

We tested whether the overall intensity of subjective effects (total VAS) related to global EEG power across all frequency bands, implementing an Analysis of Variance (ANOVA) model including EEG power in all bands and dose as predictors. This analysis was conducted for total VAS scores obtained 1, 2, 3, and 4 h after psilocybin administration. The models yielded significant results for *t* = 1 h [*F*(6,36) = 3.53, *P* = .01], 3 h [*F*(6,36) = 3.41, *P* = .012], and 4 h [*F*(6,36) = 10.57, *P* = .000001], with non-significant results for 2 h [*F*(6,36) = 1.32, *P* = .29]. Next, we implemented band-specific ANOVA models for each time point, which are summarized in Supplementary Tables S7–S10. While some models yielded significant effects of EEG power on the total VAS scores, the interactions were non-significant. This result indicates lack of evidence of a modulatory effect of psilocybin on the association between EEG power and VAS scores.

Figure 5b displays the topographic distribution of z-scored spectral power for the delta, theta, alpha, and gamma bands, as well as the broadband LZc, for both dose conditions. Additionally, topographic maps of the t-values derived from the statistical tests comparing conditions are also displayed, for the same frequency bands and complexity measure. As shown in the last column of Fig. 5b, increased LZc was found in the high-dose condition across all channels.

### Correlations between spectral power and behavioral metrics

**Figure 6:**
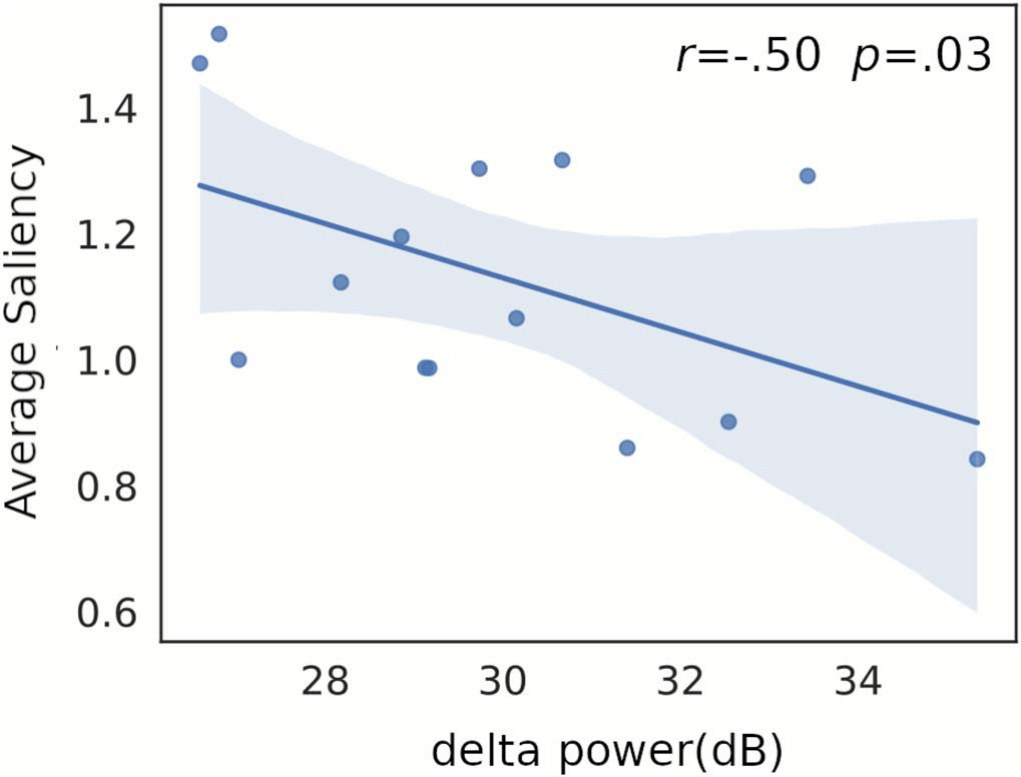
Scatter plot of the average saliency vs. delta power, including the best linear fit in the least squares sense for the high-dose condition (shaded areas represent 95% confidence intervals, Pearson’s correlation coefficient (*r*) and corresponding *P*-value are displayed in the inset).

To investigate the relationship between neural oscillatory activity and visual exploration behavior, we first implemented an ANOVA model including delta, theta, alpha, beta, and gamma EEG power and dose as predictors, providing a unified test of the main effects and interactions of interest. This omnibus model was significant [*F*(6,36) = 2.52, *P* = .039] for the prediction of the average saliency, and non-significant for the prediction of the Shannon entropy of the high saliency regions [*F*(6,36) = 1.18, *P* = .371]. Subsequently, we implemented band-specific ANOVA models. We found that only the model for delta was significant [*F*(3,24) = 4.1, *P* = .017], with a significant effect of the EEG power [*F*(1,24) = 6.13, *P* = .019] and a borderline *P*-value for the interaction between EEG power and dose [*F*(1,24) = 4.08, *P* = .051]. The other models did not yield significant results with threshold *P* = 0.05 (*F* and *P*-values for all models are provided in Supplementary Table S6). Finally, we computed correlations between the power band with a significant effect on saliency (delta) and the average saliency for both doses (Fig. 6). This analysis revealed significant negative correlations only in the high-dose condition between delta power and average saliency across all fixations. Correlation values between EEG spectral power and the saliency metric, including corrected and uncorrected *P*-values for both conditions, are provided in Supplementary Tables S4 and S5.

### Correlation between expectancy and visual salience metrics

We performed correlation analyses to examine whether individual differences in expectancy of absorption were associated with the observed changes in visual salience processing. We calculated Spearman’s rank correlations between EXP-Absorption scores and our primary outcome measures (Average Saliency and Cluster Entropy) separately for the high-dose and low-dose conditions. The analysis revealed no significant correlations, indicating that although participants reported higher EXP of absorption prior to the high-dose session, these EXP did not predict the degree of salience processing or gaze entropy between salient regions observed during the task. The results of this analysis are shown in Supplementary Table S11.

## Discussion

This study explores how psilocybin influences initial gaze behavior in natural scenes, focusing on eye-tracking metrics, and saliency processing. Our findings indicate that psilocybin induces significant alterations in gaze behavior, characterized by shorter distances between fixations and increased attention (reflected by the location of gaze fixations) toward visually salient regions. These effects are accompanied by higher entropy of the sequence of fixations in high saliency regions explored during the visual perception task. EEG analyses revealed significant dose-dependent changes in spectral power across frequency bands and LZc. These results suggest an association between resting state EEG delta power and average saliency in the high-dose condition.

The observed reduction in the average distance between fixations aligns with our previous report indicating more localized visual exploration during psychedelic states (Muller et al. 2023). Fixation durations did not differ between conditions, suggesting that participants maintained similar timing while restricting their exploration to smaller regions. We observed an increase in Shannon entropy within the sequence of high-saliency regions explored during perception. In contrast to our previous analysis conducted in Muller et al. (2023), the stimulus presentations were considerably shorter in duration, which did not allow adequate sampling of the probability distribution required to compute the global entropy. Note that this global metric differs from our entropy-across-clusters measure: even with restricted exploration (low global entropy), entropy across clusters can increase if fixations are distributed over multiple salient regions. Taken together, this pattern could reflect a tendency toward hyper-focused attention or difficulty in disengaging from visually compelling stimuli. An alternative interpretation could involve visual instability or nystagmus, for example due to motion illusion; however, the percentage of outside-screen fixations was comparable, and participants directed more fixations to the most salient regions, making this explanation more unlikely.

Altogether, these findings indicate that psilocybin alters how participants engage with salient visual regions. However, the underlying mechanisms remain open to interpretation. One possibility is that the increase in focus on salient regions reflects reduced top-down predictive control, leading to stronger influence of bottom-up sensory input. This perspective is consistent with theoretical models proposing that psychedelics relax higher-order priors, thus increasing the impact of bottom-up sensory inputs (Carhart-Harris and Friston 2019). However, we did not employ hierarchical inference modeling, manipulations of prior EXP, or predictive coding metrics to directly test this hypothesis. Beyond this interpretation, studies employing directional modeling show that psychedelic effects on hierarchical signaling are not uniform but context-dependent. In resting-state functional magnetic resonance imaging (fMRI), Stoliker et al. (2024) reported that psilocybin reduced top-down connectivity from higher-order cortical regions to the amygdala. Also in resting state, Stoliker et al. (2023) found that lysergic acid diethylamide (LSD) reorganized directed connectivity between salience, default-mode, and attention networks, indicating large-scale changes in attentional dynamics. By contrast, during a visual imagery task, Stoliker et al. (2025) observed that psilocybin enhanced feedback signals within the visual hierarchy, suggesting reinforcement of top-down pathways when participants generated visual content from imagination rather than external input. Taken together, current evidence points to context-dependent effects of psychedelics on hierarchical signaling. Our results add a behavioral perspective to this picture but cannot, on their own, identify the underlying mechanism. Future experiments to test the possibility of weakened top-down modulation under psychedelics could include the manipulation of prior EXP, together with hierarchical inference modeling and predictive coding metrics, as well as the use of neuroimaging tools with improved spatial resolution (e.g. fMRI) allowing to investigate feedforward and recurrent information processing along the visual cortical hierarchy.

Our findings differ from previous work reporting reduced neural responses to salient and novel auditory stimuli, together with enhanced responses to habitual inputs, under psychedelics (Timmermann et al. 2018). Whereas that study showed attenuated neural sensitivity to salience in the auditory domain, we observed increased allocation of gaze to salient regions during visual perception. This divergence may reflect differences across sensory modalities, measurement domains, or task contexts, and highlights the need for future studies combining behavioral and neural measures within the same paradigm.

EEG results corroborated the reports of subjective effect intensity, with significant broadband reductions in spectral power, a robust neural marker of psychedelic effects (Riba et al. 2002, Kometer et al. 2013, Muthukumaraswamy et al. 2013, Schenberg et al. 2015, Carhart-Harris et al. 2016, Valle et al. 2016, Schartner et al. 2017, Timmermann et al. 2019, Pallavicini et al. 2021). We observed a negative association between EEG delta power and the average saliency, with the interaction between power and dose close to the statistical threshold. Nevertheless, a modulatory effect of the psilocybin dose was not established due to the non-significant interaction. The negative correlations observed between delta power and the average saliency suggest an association between resting-state spectral features and visual exploration metrics, which may reflect broader state changes (e.g. vigilance, arousal) indirectly related to visual attention. This correlation analysis was exploratory. Given the number of tests and the modest EEG sample size, while none of the effects survived correction for multiple comparisons, they approached the threshold for statistical significance; we therefore report uncorrected *P*-values and caution against strong inferences. Future confirmatory studies with EEG acquired during visual perception tasks should be conducted to address the possibility of an association between visual behavior and resting state EEG oscillatory power, as well as a modulatory effect of psilocybin.

This study presents distinct advantages stemming from its naturalistic design, favoring ecological validity over controlled laboratory conditions. The self-blinding of the high vs. low doses allowed the subjects to participate in this study as part of their own natural use of the substance, resulting in a balance between the strictly controlled conditions of a standard double-blind study and the uncontrolled nature of a fully naturalistic study (Haijen et al. 2018, Sanz et al. 2022, Tagliazucchi, 2022, van Elk et al. 2022). Previous studies have demonstrated that laboratory settings are associated with increased anxiety and discomfort in subjects under moderate to high doses of serotonergic psychedelics, which may contribute towards decreasing their engagement in the tasks and negatively affect the overall quality of the recorded neural and behavioral data (Studerus et al. 2012). Psychedelics are also known to induce deeply meaningful experiences with long-lasting effects, posing ethical challenges when studying participants in artificial settings under instructions to perform monotonous and repetitive tasks.

Another advantage of our experimental design is the use of visual perception tasks that do not require active participation from the participants, as in tasks where the main outcomes are performance metrics (e.g. accuracies, reaction times). As in our previous study investigating visual behavior during esthetic perception (Muller et al. 2023), participants were instructed to passively view the stimuli as they would normally do. It has been established that psychedelics compromise different aspects of cognitive function, including focused attention to task instructions, resulting in barriers to experimental paradigms requiring active participation (Bayne and Carter 2018, Bălăeţ 2022). While the study of complex behaviors during natural tasks poses significant challenges, especially concerning data analysis, it may contribute to circumventing the limitations associated with tasks that are biased by impaired performance under the acute effects of psychedelics (Tagliazucchi 2022).

Our choices regarding the experimental protocol also present some limitations that merit careful consideration. The self-blinding protocol lacked controlled sourcing and chemical quantification of the mushroom material. While the psychedelics effects were confirmed by subjective reports and robust objective neural metrics (i.e. broadband reductions of EEG power), variability introduced by lack of control over the effective psilocybin dose cannot be discarded. Another limitation concerns participant unblinding, a persistent methodological challenge in psychedelic research protocols (van Elk et al. 2022), which is manifest even when implementing active control conditions (i.e. a low dose). However, the objective physiological nature of oculomotor metrics provides reasonable assurance that condition awareness minimally influenced the eye movement patterns observed in our study. Another consideration is the relatively small and predominantly male sample, which may limit generalizability. Finally, the exploratory nature of this study must be considered when discussing its limitations. While exploratory investigations serve a crucial function in understudied domains—particularly regarding the sensitivity of ocular metrics to psychedelic-induced behavioral alterations (Jaeger and Halliday 1998)—they inherently introduce analytical flexibility that may compromise statistical reliability. To mitigate this concern, we restricted our analytical approach to fundamental oculomotor parameters and saliency and entropy measures, guided by established theoretical frameworks including the entropic brain hypothesis and the REBUS model (Carhart-Harris and Friston 2019). Future research should build upon these preliminary findings through hypothesis-driven, pre-registered research protocols to further elucidate the observed phenomena.

In conclusion, our findings demonstrate that psilocybin significantly alters visual attention and exploration strategies, shifting behavior toward increased focus on salient regions and less predictable gaze patterns within these regions, while opening the way to future work addressing the mechanisms of psychedelic-induced changes to visual saliency processing by combining perceptual tasks with simultaneous neural recordings.

## Supplementary Material

sup_mat_re_re_sub_niaf060

## Data Availability

The raw data and stimuli are publicly available on OSF at https://osf.io/5hr7a/?view_only=971855c5c0cd447fbcbece6a9f477631.
