# Supplementary material for: Psilocybin-induced modulation of visual salience processing
Source: Neurosci Conscious. 2025 Dec 26;2025(1):niaf060. doi: 10.1093/nc/niaf060 (PMC12740852; doi:10.1093/nc/niaf060)
Supplement: sup_mat_re_re_sub_niaf060 [file sup_mat_re_re_sub_niaf060.pdf]

Supplementary material

Self-reported scales and questionnaires

Big Five Inventory (BFI). A validated Spanish version of the inventory assessing five dimensions of personality: neuroticism, extraversion, openness to experience, agreeableness, and conscientiousness (Benet-Martínez & John, 1998). The BFI questionnaire consists of 44 items based on a 5-point Likert scale. Multiple studies suggest that psychedelics are capable of inducing short and long-term changes in personality (Bouso et al., 2018). Moreover, some of these changes (e.g. increased openness) might be contributing factors to the therapeutic effects of psychedelics, as well as to the long-term positive changes in subjective well-being reported by healthy Individuals

Short Suggestibility Scale (SSS). An inventory that assesses suggestibility, created by Kotov (2004), and translated to Spanish by the authors. The questionnaire consists of 21 items based on a 5-point Likert scale. It has been shown that psychedelics can enhance suggestibility in healthy volunteers (Carhart-Harris et al., 2015)

Tellegen Absorption Scale (TAS). A 34-item scale developed to measure the capacity of an individual to become absorbed in the performance of a task (Tellegen & Atkinson, 1974), translated to Spanish by the authors. This psychological construct is positively correlated with the overall intensity of the effects elicited by psychedelic drugs, and also implicated in some of its most intriguing effects, such as the induction of spiritual or mystical experiences(Haijen et al., 2018)

State-Trait Anxiety Inventory (STAI-T / STAI-S). Validated Spanish editions of commonly used scales which measure state anxiety (situational anxiety of a temporary nature) and trait anxiety (stable trait linked to individual characteristics) (Spielberger, 1983). The instrument comprises 40 items and is based on a 4-point Likert scale.

Positive and Negative Affect Schedule (PANAS). A validated Spanish version of a psychometric scale that has been widely used to measure dimensions of affect, both positive and negative (Watson et al., 1988). The instrument consists of 20 affirmations based on a 5-point Likert scale.

Psychological Well-being Scale (BIEPS). A scale used to measure eudaemonic well-being in adults (including dimensions of acceptance, perception of control, social ties, and autonomy and projects) (Castro, 2002) It consists of 13 questions based on a 3-point Likert scale.Originally developed in Spanish.

Pre-ceremony Scale (PRE). Twelve items assessing non-pharmacological contextual factors prior to psychedelic experiences. Using principal component analysis, Haijen et al. showed that the items clustered into three components: set, setting and clear intentions. (Haijen et al., 2018).

Expectation (EXP). Seventeen questions designed to measure expectations of change in the following areas: positive emotions, negative emotions, anxiety, attention, absorption, creativity, perception, problem solving, empathy, memory, energy, sleep, sociability, spirituality, openness, oceanic feeling and substance intake (Cavanna et al., 2022).

Altered States of Consciousness (5D-ASC). Ninety-four items assessing different aspects of altered states of consciousness, understood as temporary deviations from normal waking consciousness. Consists of three main dimensions, each with several lower-order scales. The dimensions and their corresponding scales are: oceanic boundlessness (experience of oneness, spiritual experience, state of bliss, insightfulness), anxious ego dissolution

(disembodiment, impaired control and cognition, anxiety), visionary restructuration (complex images, elementary images, audiovisual synesthesia, altered meaning of perceptions) (Studerus et al., 2010).

Mystical Experiences Questionnaire (MEQ-30). Thirty items from which four subscale scores are calculated: mystical, positive mood, transcendence of time and space, and ineffability, which are considered the most relevant and defining aspects of mystical experiences (Barrett et al., 2015).

A visual analogue scale (VAS) was provided to the subjects for the purpose of rating their subjective effects on four occasions throughout the course of the experiment. This scale is an abridged version of a previously employed scale (Cavanna et al., 2022; Pallavicini et al., 2021), which is limited to items pertaining to the effects of the drug on perception. The items were rated on a visual analogue scale (VAS) comprising the following statements: "Sounds influence what I see", "My sense of size and space is distorted", "I feel unusual bodily sensations", "I see geometric patterns", "Edges seem warped", "I see movement in things that aren't really moving" and "Things look strange". The outcome of this scale is presented in Figure S1.

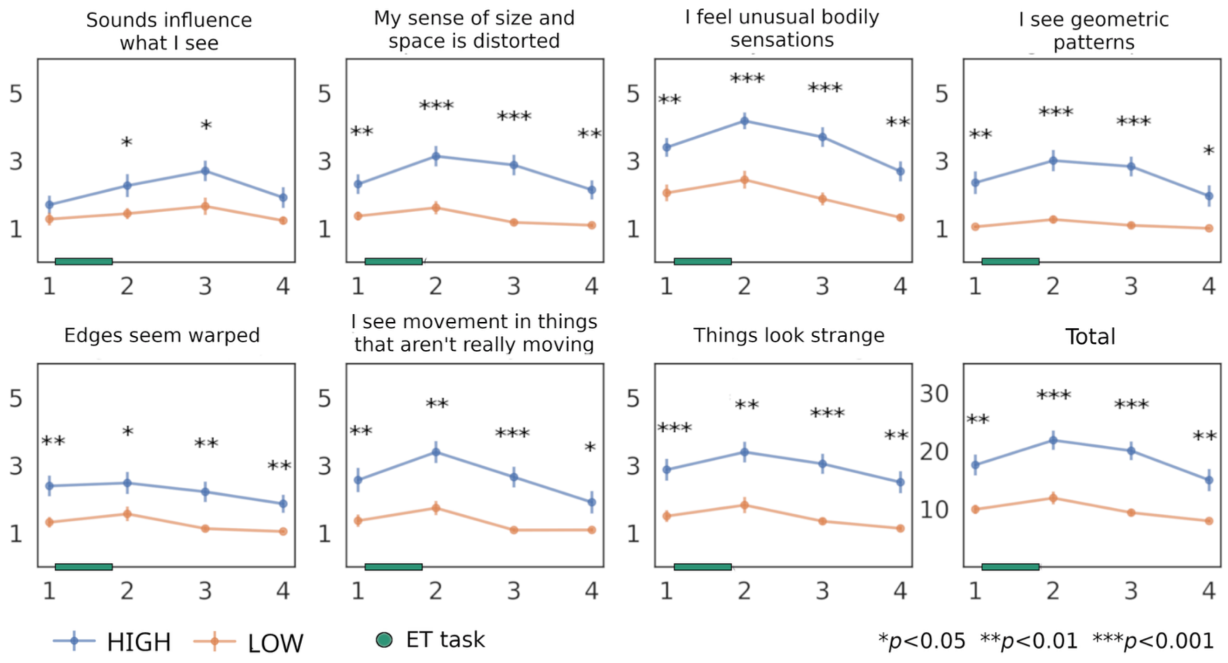

Fig S1: Acute effects measured using individual VAS items and the overall intensity of the experience given by the sum of all the items. Results are shown for each measurement time point, with consecutive measurements separated by one hour. The points indicate the mean across participants and the vertical lines the standard error of the mean (SEM). The time during which the eye tracking task (ET) took place is indicated in the x-axis. Statistical significance is indicated using asterisks (Wilcoxon signed- rank tests). *p<0.05 (Benjamini-Hochberg FDR correction).

Statistical fixation metrics

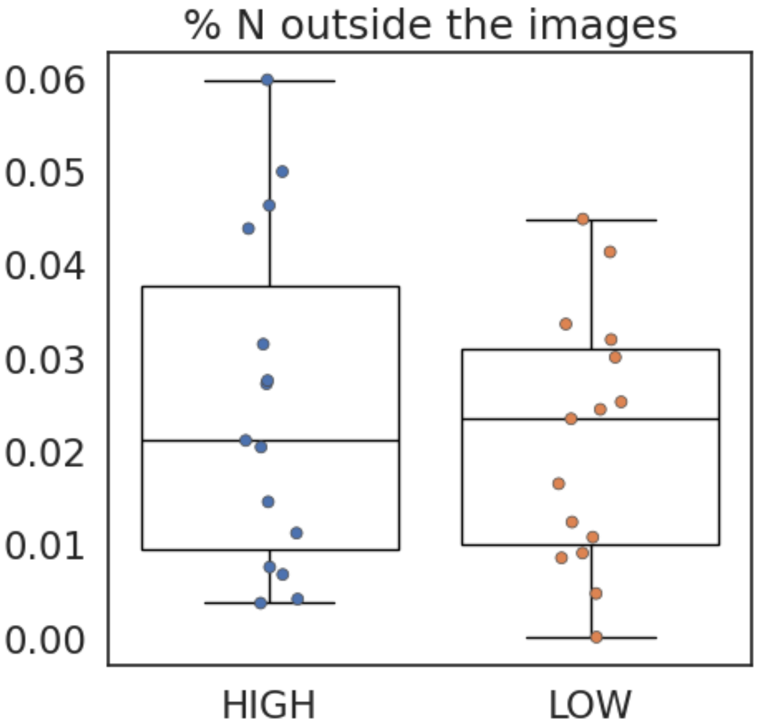

Fig S2: Percentage of fixations outside the image boundaries. Individual data points and group distributions are shown for the high-dose and low-dose conditions. Boxplots represent the interquartile range (IQR), with the median indicated by a central line; whiskers extend to 1.5 × IQR. The inset shows the p-value from a Wilcoxon signed-rank test, indicating no significant differences between conditions.

Summary of statistical analyses

The results of all the self-reported scales and questionnaires are presented in Table S1. The results of the acute effects measurements are presented in Table S2 and Table S3

Table S1.

Scale - Factor	Mean ± SD Psilocybin	Mean ± SD Placebo	p-value	Cohen's d	BF_{10}
**Baseline**					
STAI - Trait	21.04 ± 8.39	20.61 ± 8.04	645	0.05	0.24
BFI - Extraversion	28.61 ± 4.15	28.57 ± 3.80	945	0.01	0.22
BFI - Agreeability	34.26 ± 4.85	34.48 ± 4.87	623	0.04	0.25
BFI - Conscientiousness	30.96 ± 5.47	30.00 ± 6.54	0.28	0.16	0.38
BFI - Neuroticism	21.78 ± 7.39	20.87 ± 6.36	166	0.13	0.54
BFI - Openness	43.48 ± 4.59	42.83 ± 5.58	303	0.13	0.36
TAS	21.83 ± 6.14	20.57 ± 6.73	158	0.2	0.56
SSS	56.48 ± 11.24	53.26 ± 10.21	77	0.3	0.94
**Pre dose**					
STAI - State	11.26 ± 8.62	12.09 ± 8.38	692	0.1	0.23
PANAS - Negative affect	15.32 ± 5.16	14.09 ± 4.46	274	0.26	0.38
PANAS - Positive affect	32.57 ± 9.31	34.30 ± 7.86	268	0.2	0.39
BIEPS	35.48 ± 2.87	35.55 ± 2.86	875	0.02	0.22
PRE - Set	6.63 ± 0.66	6.60 ± 0.79	861	0.05	0.22
PRE - Setting	8.54 ± 1.10	8.49 ± 1.50	816	0.03	0.22
PRE - Clear intentions	5.74 ± 1.96	5.67 ± 2.87	839	0.03	0.22
EXP - Negative emotions	2.00 ± 0.80	2.00 ± 0.80	1000	0	0.22
EXP - Anxiety	1.91 ± 0.90	2.04 ± 1.02	623	0.14	0.25
EXP - Attention	3.13 ± 1.32	3.30 ± 1.18	505	0.14	0.27
**EXP - Absorption**	**4.17 ± 0.72**	**3.74 ± 0.54**	**0.009 ****	**0.68**	**5.36**
EXP - Creativity	4.17 ± 0.58	3.96 ± 0.77	171	0.32	0.53
EXP - Perception	4.50 ± 0.66	4.26 ± 0.62	102	0.37	0.77
EXP - Problem resolution	3.26 ± 1.39	3.35 ± 1.07	704	0.07	0.23
EXP - Empathy	4.39 ± 0.58	4.13 ± 0.81	56	0.37	1.21
EXP - Memory	2.91 ± 1.12	3.09 ± 0.95	406	0.17	0.30
EXP - Energy	3.74 ± 1.10	3.61 ± 0.89	575	0.13	0.25
EXP - Sleep	2.74 ± 1.01	2.61 ± 0.89	503	0.14	0.27
**EXP - Sociability**	**3.83 ± 0.94**	**3.35 ± 1.07**	**0.038 (*)**	**0.48**	**1.64**
EXP - Spirituality	4.14 ± 0.69	3.96 ± 0.71	204	0.26	0.47
EXP - Openness	4.39 ± 0.78	4.13 ± 0.69	162	0.35	0.55

**EXP - Oceanic feelings**	**4.61 ± 0.58**	**4.17 ± 0.72**	**5e-4 (***)**	**0.67**	**70.52**
EXP - Substance intake	2.65 ± 0.83	2.83 ± 0.78	406	0.22	0.30
EXP - Total	60.57 ± 5.69	58.61 ± 6.46	108	0.32	0.73
**Post dose**					
STAI - State	10.79 ± 7.97	8.43 ± 7.25	128	0.31	0.65
**PANAS - Negative affect**	**16.23 ± 5.43**	**14.26 ± 5.82**	**108**	**0.35**	**0.73**
PANAS - Positive affect	32.68 ± 9.94	34.61 ± 7.77	277	0.22	0.38
BIEPS	8.41 ± 0.89	8.57 ± 0.73	314	0.19	0.35
**MEQ - Mystical experience**	**21.38 ± 15.48**	**4.48 ± 6.49**	**4e-6 (***)**	**1.42**	**4565**
**MEQ - Positive mood**	**14.41 ± 6.34**	**6.52 ± 4.39**	**4e-5 (***)**	**1.45**	**686**
**MEQ - Transcendence**	**12.45 ± 5.44**	**2.35 ± 2.57**	**2e-7 (***)**	**2.38**	**91510**
**MEQ - Ineffability**	**8.23 ± 3.67**	**1.57 ± 1.70**	**3e-8 (***)**	**2.33**	**435700**
**5DASC - Unity**	**14.83 ± 11.28**	**6.35 ± 1.97**	**0.001 (**)**	**1.05**	**31.07**
**5DASC - Spiritual**	**8.13 ± 5.29**	**4.13 ± 2.34**	**0.003 (**)**	**0.98**	**15.71**
**5DASC - Blissful**	**12.23 ± 8.18**	**5.39 ± 3.14**	**0.001 (***)**	**1.1**	**53.24**
**5DASC - Insightfulness**	**12.59 ± 8.34**	**4.91 ± 3.80**	**6e-5 (***)**	**1.18**	**454**
**5DASC - Disembodiment**	**7.27 ± 5.55**	**3.57 ± 1.50**	**0.003 (**)**	**0.91**	**13.77**
**5DASC - Impaired cognition**	**22.86 ± 10.48**	**8.57 ± 3.01**	**1e-6 (***)**	**1.86**	**15920**
**5DASC - Anxiety**	**17.50 ± 9.70**	**7.26 ± 3.02**	**9e-5 (***)**	**1.42**	**294**
**5DASC - Complex imagery**	**15.73 ± 9.10**	**4.30 ± 2.98**	**2e-6 (***)**	**1.69**	**8747**
**5DASC - Elementary imagery**	**20.27 ± 10.22**	**4.57 ± 3.86**	**6e-7 (***)**	**2.03**	**30910**
**5DASC - Audio-visual syn.**	**13.41 ± 11.65**	**4.65 ± 3.95**	**0.002 (**)**	**1.01**	**24.12**
**5DASC - Changed meaning**	**14.36 ± 7.85**	**4.13 ± 2.20**	**1e-6 (***)**	**1.78**	**13850**

Table S1. Baseline, Pre dose, Acute effects and Post dose outcomes are presented as mean ± standard deviation. Statistical significance is indicated using asterisks and computed using Student's t-test.

Table S2.

Scale - Factor	Mean ± SD Psilocybin	Mean ± SD Placebo	p-value	RBC's r
**Acute Effects**				
**VAS 1 total**	**17.56 ± 8.47**	**9.91 ± 3.80**	**0.002 ****	**0.80**
**VAS 2 total**	**21.83 ± 7.79**	**11.87 ±5.19**	**0.0001*****	**0.84**
**VAS 3 total**	**20.00 ± 7.56**	**9.35 ± 2.76**	**8e-5 *****	**0.96**
**VAS 4 total**	**14.95 ± 8.00**	**7.91 ± 1.35**	**0.001 ****	**0.87**
N	9.18 ±2.18	8.59 ±1.26	0.45	0.38
**ds**	**131.48 ±19.91**	**146.37 ±16.14**	**0.041***	**-0.60**
dt	0.35 ± 0.07	0.37±0.05	0.49	-0.22
**Average saliency**	**1.14 ± 0.22**	**1.00 ± 0.11**	**0.035***	**0.62**

Table S2. Acute effects outcomes are presented as mean ± standard deviation.

Table S3.

	**Average Saliency Interval**	**p_value**	**corrected_p_value**	**RBC's r**
**0**	t1 [0,600)ms	0.36	0.36	0.28
**1**	**t2 [600,1200)ms**	**0.026**	**0.059**	**0.65**
**2**	**t3 [1200,1800)ms**	**0.035**	**0.059**	**0.62**
**3**	**t4 [1800,2400)ms**	**0.026**	**0.059**	**0.65**
**4**	t5 [2400,3000)ms	0.055	0.069	0.57

Table S3. Average saliency values compared between high-dose and low-dose conditions across the five equal 600 ms intervals of the 3-second viewing period. The table reports uncorrected p-values from Wilcoxon signed-rank tests and the corresponding FDR-corrected values.

Table S4.

	**p value**	**corrected p value**	**Pearson's R**
**Avg. saliency - delta**	**0.03**	**0.06**	**-0.50**
Avg. saliency - theta	0.08	0.21	-0.38
Avg. saliency - alpha	0.12	0.37	-0.32
Avg. saliency - beta	0.07	0.21	-0.41
**Mean saliency - delta**	**0.03**	**0.06**	**-0.50**
Mean saliency - theta	0.20	0.20	-0.24
Mean saliency - alpha	0.37	0.38	-0.09
Mean saliency - beta	0.19	0.20	-0.24

Table S4. Pearson's R values, and FDR-corrected and uncorrected p-values for the correlations between saliency metrics and EEG spectral power (high-dose condition).

Table S5.

	**p value**	**corrected p value**	**Pearson's R**
Avg. saliency - delta	0.47	0.69	-0.02
Avg. saliency - theta	0.41	0.57	-0.06
Avg. saliency - alpha	0.52	0.60	0.01
Avg. saliency - beta	0.67	0.76	0.13
Mean saliency - delta	0.69	0.69	0.15
Mean saliency - theta	0.57	0.57	0.05
Mean saliency - alpha	0.60	0.60	0.08
Mean saliency - beta	0.76	0.76	0.21

Table S5. Pearson's R values, and FDR-corrected and uncorrected p-values for the correlations between saliency metrics and EEG spectral power (for the low-dose condition).

Table S6.

Frequency band	Main effect	Effect of EEG power	Interaction dose-power
**Delta**	F(3,24)=4.1 , p=0.017	F(1,24)=6.13 , p=0.019	F(1,24)=4.08 , p=0.051
**Theta**	F(3,24)=2.98 , p=0.052	F(1,24)=3.45 , p=0.075	F(1,24)=2.07 , p=0.16
**Alpha**	F(3,24)=2.52 , p=0.081	F(1,24)=2.34 , p=0.138	F(1,24)=1.18 , p=0.285
**Beta**	F(3,24)=2.56 , p=0.078	F(3,24)=2.56 , p=0.078	F(3,24)=2.56 , p=0.078
**Gamma**	F(3,24)=2.15 , p=0.12	F(3,24)=2.37 , p=0.14	F(3,24)=2.06, p=0.15

Table S6. Main effect, effect of EEG power and interaction dose-power for ANOVA models of saliency (saliency~power*dose).

Table S7.

Frequency band	Main effect	Effect of EEG power	Interaction dose-power
**Delta**	F(3,24)=4.79 , p=0.009	F(1,24)=8.12, p=0.009	F(1,24)=0.51 , p=0.47
**Theta**	F(3,24)=1.23 , p=0.318	F(1,24)=0.58 , p=0.45	F(1,24)=0.005 , p=0.94
**Alpha**	F(3,24)=1.58 , p=0.22	F(1,24)=1.16 , p=0.29	F(1,24)=2.46 , p=0.13
**Beta**	F(3,24)=1.93 , p=0.15	F(3,24)=0.72 , p=0.41	F(3,24)=0.36 , p=0.55
**Gamma**	F(3,24)=7.54 , p=0.001	F(3,24)=12.78, p=0.002	F(3,24)=0.10 , p=0.75

Table S7. Main effect, effect of EEG power and interaction dose-power for ANOVA models of total VAS after 1 hour (VAS~power*dose).

Table S8.

Frequency band	Main effect	Effect of EEG power	Interaction dose-power
**Delta**	F(3,24)=2.42 , p=0.09	F(1,24)=1.11, p=0.17	F(1,24)=0.26 , p=0.61
**Theta**	F(3,24)=1.66 , p=0.20	F(1,24)=0.11 , p=0.73	F(1,24)=0.003 , p=0.95
**Alpha**	F(3,24)=1.65 , p=0.20	F(1,24)=0.43 , p=0.51	F(1,24)=0.09 , p=0.72
**Beta**	F(3,24)=1.62 , p=0.21	F(3,24)=0.0006, p=0.98	F(3,24)=0.18 , p=0.67
**Gamma**	F(3,24)=2.95, p=0.51	F(3,24)=1.69, p=0.20	F(3,24)=0.32, p=0.57

Table S8. Main effect, effect of EEG power and interaction dose-power for ANOVA models of total VAS after 2 hours (VAS~power*dose).

Table S9.

Frequency band	Main effect	Effect of EEG power	Interaction dose-power
**Delta**	F(3,24)=8.4 , p=0.0005	F(1,24)=4.27, p=0.50	F(1,24)=1.17 , p=0.28
**Theta**	F(3,24)= 5.94, p=0.003	F(1,24)=0.21 , p=0.64	F(1,24)=0.06 , p=0.79
**Alpha**	F(3,24)=6.69 , p=0.001	F(1,24)= 1.53, p=0.22	F(1,24)=0.94 , p=0.33
**Beta**	F(3,24)=6.01 , p=0.003	F(3,24)=0.02, p=0.87	F(3,24)=0.27 , p=0.60
**Gamma**	F(3,24)=6.24, p=0.002	F(3,24)=0.12, p=0.72	F(3,24)=0.75, p=0.38

Table S9. Main effect, effect of EEG power and interaction dose-power for ANOVA models of total VAS after 3 hours (VAS~power*dose).

Table S10.

Frequency band	Main effect	Effect of EEG power	Interaction dose-power
**Delta**	F(3,24)=3.33 , p=0.036	F(1,24)=1.50, p=0.23	F(1,24)=0.38 , p=0.54
**Theta**	F(3,24)= 2.68 , p=0.06	F(1,24)=7.18 , p=0.013	F(1,24)=0.004 , p=0.94
**Alpha**	F(3,24)=5.87 , p=0.003	F(1,24)= 1.53, p=0.22	F(1,24)=4.53 , p=0.043
**Beta**	F(3,24)=3.44 , p=0.03	F(3,24)=1.66, p=0.20	F(3,24)=1.41 , p=0.24
**Gamma**	F(3,24)=3.52, p=0.03	F(3,24)=1.79 , p=0.19	F(3,24)=1,02 , p=0.28

Table S10. Main effect, effect of EEG power and interaction dose-power for ANOVA models of total VAS after 4 hours (VAS~power*dose).

Table S11.

	High-dose		Low-dose	
	Spearman rho	Spearman p	Spearman rho	Spearman p
Average Saliency	0.205	0.463	-0.360	0.187
Cluster Entropy	-0.120	0.671	-0.065	0.817

Table S11. Spearman correlation coefficients and p-values between the Expectancy of Absorption (EXP-Absorption) scores and the main visual saliency metrics (Average Saliency and Cluster Entropy) for both high-dose and low-dose conditions.

References

Barrett, F. S., Johnson, M. W., & Griffiths, R. R. (2015). Validation of the revised mystical experiences questionnaire in experimental sessions with psilocybin. *Drug and Alcohol Dependence*, *156*, e16. https://doi.org/10.1016/j.drugalcdep.2015.07.961

Benet-Martínez, V., & John, O. P. (1998). Los Cinco Grandes across cultures and ethnic groups: Multitrait-multimethod analyses of the Big Five in Spanish and English. *Journal of Personality and Social Psychology*, *75*(3), 729–750. https://doi.org/10.1037/0022-3514.75.3.729

Bouso, J. C., dos Santos, R. G., Alcázar-Córcoles, M. Á., & Hallak, J. E. C. (2018). Serotonergic psychedelics and personality: A systematic review of contemporary research. *Neuroscience & Biobehavioral Reviews*, *87*, 118–132. https://doi.org/10.1016/j.neubiorev.2018.02.004

Carhart-Harris, R. L., Kaelen, M., Whalley, M. G., Bolstridge, M., Feilding, A., & Nutt, D. J. (2015). LSD enhances suggestibility in healthy volunteers. *Psychopharmacology*, *232*(4), 785–794. https://doi.org/10.1007/s00213-014-3714-z

Castro, A., Brenlla, M. ,. &. Casullo, M. (2002). *Evaluación del bienestar psicológico en iberoamérica*.

Cavanna, F., Muller, S., de la Fuente, L. A., Zamberlan, F., Palmucci, M., Janeckova, L., Kuchar, M., Pallavicini, C., & Tagliazucchi, E. (2022). Microdosing with psilocybin mushrooms: A double-blind placebo-controlled study. *Translational Psychiatry*, *12*(1).

https://doi.org/10.1038/s41398-022-02039-0

Haijen, E. C. H. M., Kaelen, M., Roseman, L., Timmermann, C., Kettner, H., Russ, S., Nutt, D., Daws, R. E., Hampshire, A. D. G., Lorenz, R., & Carhart-Harris, R. L. (2018). Predicting Responses to Psychedelics: A Prospective Study. *Frontiers in Pharmacology*, *9*. https://doi.org/10.3389/fphar.2018.00897

Kotov, R. I., Bellman, S. B. ,. &. Watson, D. B. (2004). *Short Suggestibility Scale.*

Pallavicini, C., Cavanna, F., Zamberlan, F., de la Fuente, L. A., Ilksoy, Y., Perl, Y. S., Arias, M., Romero, C., Carhart-Harris, R., Timmermann, C., & Tagliazucchi, E. (2021). Neural and subjective effects of inhaled N,N-dimethyltryptamine in natural settings. *Journal of Psychopharmacology*, *35*(4), 406–420. https://doi.org/10.1177/0269881120981384

Spielberger, C. D. (1983). *State-trait anxiety inventory for adults.*

Studerus, E., Gamma, A., & Vollenweider, F. X. (2010). Psychometric evaluation of the altered states of consciousness rating scale (OAV). *PLoS ONE*, *5*(8). https://doi.org/10.1371/journal.pone.0012412

Tellegen, A., & Atkinson, G. (1974). Openness to absorbing and self-altering experiences (“absorption”), a trait related to hypnotic susceptibility. *Journal of Abnormal Psychology*, *83*(3), 268–277. https://doi.org/10.1037/h0036681

Watson, D., Clark, L. A., & Carey, G. (1988). Positive and negative affectivity and their relation to anxiety and depressive disorders. *Journal of Abnormal Psychology*, *97*(3), 346–353. https://doi.org/10.1037/0021-843X.97.3.346
